# Conjunctional Relationship between Serum Uric Acid and Serum Nickel with Non-Alcoholic Fatty Liver Disease in Men: A Cross-Sectional Study

**DOI:** 10.3390/ijerph19116424

**Published:** 2022-05-25

**Authors:** Chili Liu, Wannian Liu, Guofu Zhang, Yongbin Wang, Jing Jiang, Zhongzhi Yang, Weidong Wu

**Affiliations:** School of Public Health, Xinxiang Medical University, Xinxiang 453003, China; liuchili7777777@126.com (C.L.); nian88898@126.com (W.L.); zgfxxmu@163.com (G.Z.); wybwho@163.com (Y.W.); jiangjing2019@sina.com (J.J.)

**Keywords:** non-alcoholic fatty liver disease, serum uric acid, serum nickel, risk factor, dose response relationship, cross-sectional study

## Abstract

Serum uric acid (SUA) and heavy metals are closely related to non-alcoholic fatty liver disease (NAFLD). Yet, the conjunctional relationship between SUA and serum nickel (Ni) concentrations with the risk of NAFLD in men has not yet been investigated. Therefore, we designed this cross-sectional study to investigate the association of SUA or serum Ni with NAFLD in men. The cross-sectional study was based on data obtained from a prospective cohort study of common chronic non-communicable diseases in Central China, conducted in Xinxiang city, Central China’s Henan Province, between April and June 2017. A total of 1709 male participants completed the physical examination. B-ultrasound was used to examine the liver and to diagnose NAFLD. Binary logistic regression models and restricted cubic splines were performed to estimate the association of the SUA and serum Ni with NAFLD. The prevalence of NAFLD among 1709 male participants was 46.6%. After adjusting for potential confounders, with the highest quartile compared to those with the lowest quartile, SUA (OR = 1.579, 95% CI: 1.140–2.189) and serum Ni (OR = 1.896, 95% CI: 1.372–2.625) were associated with NAFLD, respectively. At the same time, the associations for the second and third SUA quartiles were null. Restricted cubic splines showed a positive linear relationship between serum Ni (ln-transformed) and NAFLD risk. Intriguingly, high SUA and high Ni (OR = 2.370, 95% CI: 1.577–3.597) increased the risk of NAFLD, compared with those with low SUA and low Ni. Our findings demonstrate a positive linear trend between serum Ni concentrations and NAFLD risk. Men with elevated serum Ni had a higher risk of developing NAFLD when compared to those with high SUA. Furthermore, the conjunctional relationship of SUA and serum Ni with NAFLD risk was observed in men.

## 1. Introduction

Nonalcoholic fatty liver disease (NAFLD) is a liver condition affecting people who drink little to no alcohol. It is characterized by intracellular fat accumulation. If not treated properly, it can lead to hepatocellular inflammation, fibrosis, and hepatocellular carcinoma [1]. The prevalence of NAFLD in western countries is about 20–30% [2], while in China, It is 15–30% [3], the prevalence is expected to increase from 6 to 20% by 2030 [4]. Moreover, epidemiological data have indicated a higher prevalence in males than in females [5]. NAFLD was generally considered a predominantly hepatic manifestation of metabolic syndrome. The pathological process of NAFLD has been associated with insulin resistance, lipid metabolism disorder, and inflammatory stimulation [6]. Previous studies have shown that insulin resistance, diabetes, obesity, oxidative stress, hyperuricemia, and the accumulation of heavy metals increase the risk of NAFLD [7,8,9]. Nevertheless, the exact pathogenesis is not fully understood.

Previous studies have revealed that hyperuricemia is associated with Type 2 diabetes [10], hypertension [11], and metabolic syndrome [12]. In addition, hyperuricemia may induce hepatic lipid accumulation by inducing mitochondrial oxidative stress [13] and exacerbating insulin resistance [14] or promoting inflammation [15], which in turn leads to NAFLD. In 2002, an Italian study reported that higher serum uric acid (SUA) levels in NAFLD patients than those without fatty liver [16]. Moreover, other studies successively reported a positive relationship between SUA and NAFLD [10,17]. In addition, some researchers suggested that high levels of SUA are associated with a higher risk of NAFLD in women than in men [18], while others indicated that SUA was associated with NAFLD in male Type 2 diabetic subjects [19]. Thus, further studies are needed to explore whether a high SUA level increases the rates of NAFLD among men.

With the rapid development of industrialization, human exposure to heavy metals from air, soil, water, and food pollutants have been increasing, as absorbed through the skin, digestive tract, or by breathing into the body; one of these pollutants includes nickel (Ni) [20,21,22]. High Ni results in a series of health problems, such as cardiovascular disease, hypersensitivity, and carcinogenicity [23]. Hence, among a large number of metals, Ni needs to be paid more attention for polluting the environment and threatening human health [24]. Several studies have also shown that Ni also causes hepatotoxicity [25,26]. To the best of our knowledge, no studies have explored the impact of Ni on NAFLD in men, especially the conjunctional relationship between SUA and serum Ni with NAFLD. Considering that SUA, serum Ni, and NAFLD have all been closely associated with metabolic syndrome or oxidative stress [12,27,28], we hypothesized a potential association between SUA and serum Ni with NAFLD. Therefore, we designed this cross-sectional study to investigate the association of SUA or serum Ni with NAFLD in men.

## 2. Materials and Methods

### 2.1. Study Design and Subjects

**Figure 1:**
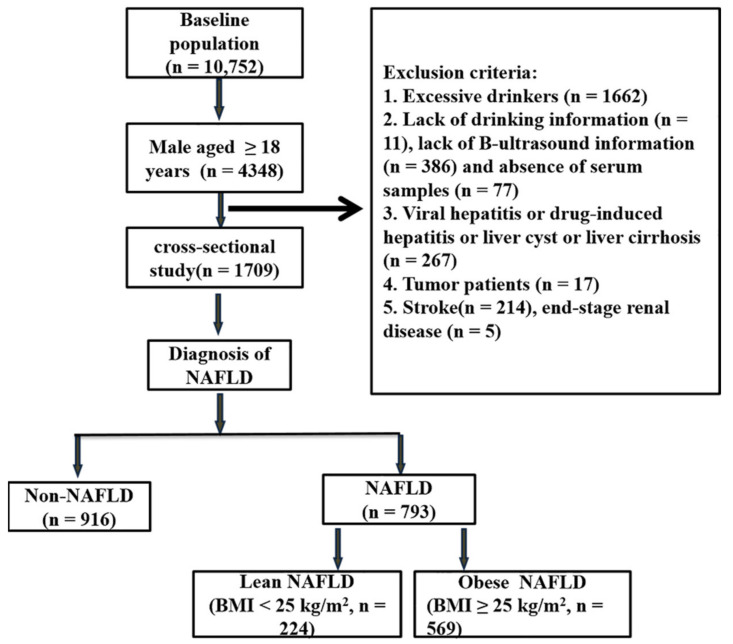
The flow diagram of study design. NAFLD, Nonalcoholic fatty liver disease; lean NAFLD, non-alcoholic fatty liver disease patients with body mass index <25 kg/m^2^; Obese NAFLD; non-alcoholic fatty liver disease patients with body mass index ≥25 kg/m^2^.

The cross-sectional study was based on data obtained from a prospective cohort study of common chronic non-communicable diseases in Central China conducted in Xinxiang city, Central China’s Henan Province, from April to June 2017. The detailed information for this cohort study has been previously reported [29]. The multistage stratified cluster sampling method has been used to obtain the general population samples from 5 areas of Xinxiang City (Suiping County, Yuzhou County, Xinxiang County, Tongxu County, and Yima County) in Henan Province, China. Finally, we identified 22 rural communities in Xinxiang County [30]. All recruited participants signed informed consent, underwent a health check-up by Xinxiang Medical University Health Team and provided detailed questionnaires. Eligibility criteria were: (1) those aged ≥18 years; (2) drinking <30 g of alcohol per day; (3) those who completed the questionnaires; (4) available data regarding, biochemical parameters, abdominal ultrasound, and serum metal determination. Those with viral hepatitis; drug-induced hepatitis; liver cirrhosis; tumor; stroke; end-stage renal disease were excluded. The flow diagram of our study design is shown in Figure 1. All participants signed the informed consent form. The study was conducted in accordance with the Declaration of Helsinki, and the protocol was approved by the Ethics Committee of Xinxiang Medical University for Human Studies (IRB number XY-HS04).

### 2.2. Data Collection and Definitions

The sociodemographic characteristics such as height, weight, age, smoking, drinking, and diet were obtained via face-to-face interviews. Smoking in the questionnaire of this study was defined as never smoking, quitting smoking, and current smoking; alcohol consumption was defined as never drinking, stopped drinking, and current drinking; meat (pork, chicken, and duck) and vegetables were non-eating, daily, weekly, monthly and annually. According to the Chinese Dietary Guidelines [31], meat consumption was defined as follows: >75 g/d for a high meat diet, <40 g/d was a low-meat diet, 40–75 g/d was a normal diet, and the vegetables consumption was defined as follows: >500 g/d was a high fiber diet, <250 g/d was a low fiber diet, 250–500 g/d was a normal diet. Biochemical index included serum uric acid (SUA), alanine aminotransferase (ALT), aspartate aminotransferase (AST), alkaline phosphatase (ALP), total bilirubin (TBIL), systolic blood pressure (SBP), diastolic blood pressure (DBP), triglyceride (TG), total cholesterol (TC), high-density lipoprotein cholesterol (HDL-C), low-density lipoprotein cholesterol (LDL-C), glycated hemoglobin (HbA_1C_), and fasting plasma glucose (FPG). These indicators were assessed by an immunochemical-automated analyzer (Type Cobas c501, Roche, Basel, Switzerland, Only HbA_1C_ was used VARIANT, Bio-Rad, Hercules, CA, USA) in the Xinxiang Medical University Health Team. Body mass index (BMI) was calculated (kg/m^2^) based on height and weight; Lean NAFLD was defined as NAFLD with BMI < 25 kg/m^2^; obese NAFLD was defined as NAFLD with BMI ≥25 kg/m^2^ [32]. Hyperuricemia was defined as: SUA (H_SUA) ≥ 420 µmol/L; SUA < 420µmol/L was considered a low SUA (L_SUA); the SUA quartiles were as follows: <275, 275–322, 322–379, ≥379 µmol/L [33]; some studies set the reference range of blood nickel as 1.6–4.6 µg/L, which was close to the average concentration of normal people [34], and some studies believed that the upper limit of blood nickel was 1.1 µg/L [35]. In this study, the average concentration of serum nickel was 4.21 µg/L, and the median concentration was 2.53 µg/L. Therefore, we regard the median concentration of 2.53 µg/L as the cut-off point between high nickel and low nickel. Hence, serum nickel ≥ 2.53 µg/L was defined as high serum nickel (H_Ni); <2.53 µg/L was defined as low serum nickel (L_Ni). The Ni quartiles were as follows: <2.03, 2.03–2.53, 2.53–3.53 and ≥3.53 µg/L. Inductive-coupled plasma mass spectrometry (iCAP Qc, Thermo Fisher Scientific, Hanna-Kunath-Strasse 11, 28199 Bremen, Germany) was used to measure serum levels of nickel. All participants agreed to abdominal B-ultrasonography examinations.

### 2.3. Diagnosis of NAFLD

Hepatic ultrasound examination (type: HITACHI HI VISION Avius ultrasound machine with a 1.0- and 5.0-MHz detector.) was performed by well-trained sonographers who were licensed as physicians and blinded to the clinical and laboratory data. B-ultrasound was used to examine the liver and to diagnose NAFLD based on the following criteria. B-ultrasound diagnostic criteria in the guidelines of Hepatology branch of the Chinese Medical Association in 2018 [36]: ① enhanced anterior field echo of the liver (bright liver); ② far- field echo attenuation of the liver; ③ the intrahepatic duct structure was not clearly displayed. The diagnosis was confirmed if two of the above three items were met and other causes that lead to hepatic steatosis, such as excessive drinking history, were excluded.

### 2.4. Assessment of Serum Ni

The serum samples were taken out of the −80 °C refrigerator and placed in the 4 °C refrigerator overnight and slowly thawed. After thawing, the mid-level serum samples were vortex collected at room temperature (20–25 °C) and diluted 25 times with 1.0% (*v*/*v*) nitric acid diluent containing 0.04% TritonX-100 original solution, 20 ppb internal standard, and 4% methanol, and then samples were subjected to ultrasound for 5 min, centrifugation at 5000 rpm for 5 min, and 1 mL of the middle-treated serum sample was taken and placed in the inductively coupled plasma mass spectrometer (ICP-MS) (iCAP Qc, Thermo Fisher Scientific, Hanna-Kunath-Strasse 11, 28199 Bremen, Germany) for detection. We implemented strict laboratory quality control; two external standard reference samples (SeronormTM, Stasjonsveien 44, NO-1396 Billingstad, Norway, Trace Elements Serum l-1 (l-2), LOT1309438 (LOT1309416)) were used for the quality control of 22 metal elements to be tested, including a nickel control. That is, two external quality tests per 25 samples. The recoveries ranged from 90 to 110%. The limit of detection (LOD) value of Ni was 0.00625 μg/L, and the concentration of nickel in all samples was higher than the LOD.

### 2.5. Statistical Analysis

Continuous variables with non-normal distributions were expressed as medians and interquartile ranges (IQRs). Categorical variables were described as percentages (%). The comparison between the two groups was performed using the nonparametric Mann–Whitney U-test; the Chi-squared test was used to compare the categorical data between groups.

Due to the non-normal distribution of SUA and serum Ni concentrations in males, SUA and Ni were categorized in quartiles, and the lowest concentrations were used as a control group. A binary logistic regression model was used to estimate the odds radio (OR) and 95% confidence intervals (CI) between SUA and serum Ni concentrations and NAFLD. Multifactor logistic regression was used to analyze the comparison of SUA and Ni with different NAFLD subgroups. Model 1 was adjusted for age and BMI; Model 2 was further adjusted for SBP, DBP, AST, TBIL, ALP, TC, TG, LDL-C, HDL-C, fasting glucose, HbA_1C_, meat consumption, and vegetable consumption. Restricted cubic splines (RCS) were applied to explore the dose–response relationship between SUA and serum Ni (ln-transformed) with NAFLD. RCS models had four knots at the 35th, 65th, and 95th percentiles of its distribution; the reference value (odds ratio = 1) was set at the 5th percentile. All statistical analyses were conducted using the R software (Robert Gentleman and George Ross Ihaka, University of Auckland) package version 4.2.1. *p*-value < 0.05 was considered to be statistically significant.

## 3. Results

### 3.1. Baseline Characteristics of Men Participants

The baseline characteristics of all participants are shown in Table 1. There were 1709 male participants, among whom 793 participants had NAFLD (median age of 53 years) while 916 participants were without NAFLD (median age of 58.5 years). The overall prevalence of NAFLD was 46.4% (793/1709), the median age was 55 (45, 65) years, and the median concentration of serum Ni was 2.5 µg/L. When compared with the non-NAFLD group, the participants with NAFLD were younger and overweight. Overall, the prevalence rates in 18~44 years old, 45~59 years old, and ≥60 years old were 53.7, 49.6, and 60.2% respectively. The level of SBP, DBP, ALT, ALP, SUA, serum Ni, total cholesterol, triglycerides, LDL-C, fasting glucose, and HbA_1C_ were significantly higher in the NAFLD group than in the non-NAFLD group (all *p* < 0.05), while there were no significant differences in TBIL (Table 1).

### 3.2. Basic Characteristics after Quartile of SUA and Serum Nickel

When compared with participants with lower SUA quartile (SUA_Q1), those in quartile four (SUA_Q4) tended to be younger as well as having a higher BMI, total cholesterol, triglycerides, ALT, AST, and serum nickel (all *p* < 0.001). There were no significant differences in SBP, DBP, LDL-C, TBIL, and fasting glucose in the SUA quartile groups. There were no significant differences in SBP, DBP, LDL-C, TBIL, and fasting glucose in the SUA quartile groups. Details of the baseline characteristics of participants are shown in Table S1.

With the increase in serum nickel concentrations, BMI, total cholesterol, triglycerides, ALT, and SUA all increased, while the HbA_1C_ decreased. Also, the patient with increased serum Ni concentrations tended to be younger (all *p* < 0.05). In the serum Ni quartile groups, there were no significant differences in DBP, total cholesterol, LDL-C, AST, ALP, TBIL, and fasting glucose. Details of the baseline characteristics of the participants are provided in Table S2.

### 3.3. Binary Logistic Regression Analysis of the Association between SUA and Serum Ni with NAFLD

**Figure 2:**
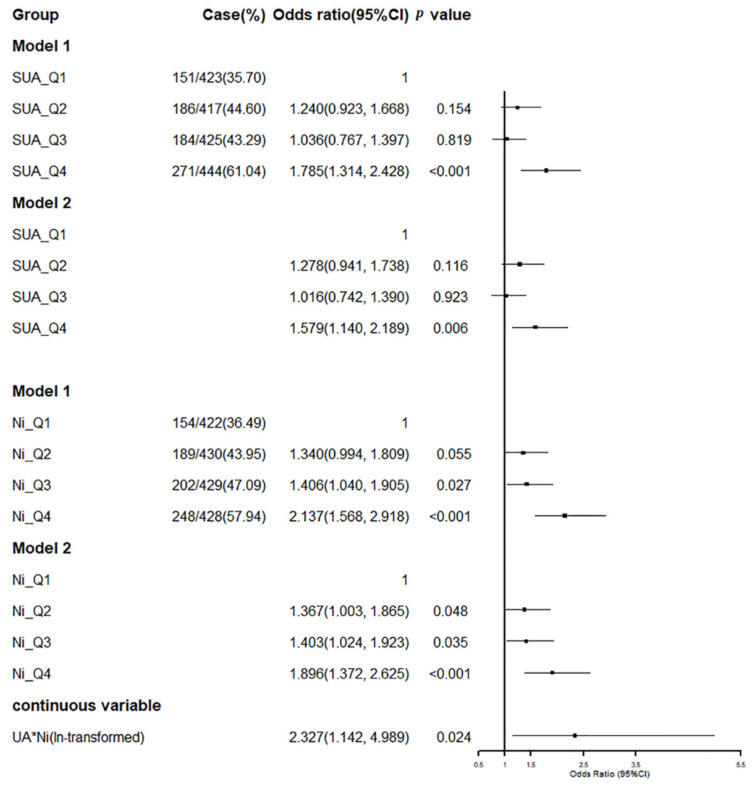
Binary logistic regression analysis of the association between SUA and serum Ni with NAFLD. The SUA quartiles were as follows: <275, 275–322, 322–379 and ≥379 µmol/L. The serum Ni quartiles were as follows: <2.03, 2.03–2.53, 2.53–3.53 and ≥3.53 µg/L. Model 1: adjusted for age and BMI; Model 2: further adjusted for SBP, DBP, ALT, AST, ALP, TBIL, TG, LDL-C, HDL-C, fasting glucose, HbA_1C_, meat consumption, and vegetable consumption. UA*Ni (ln-transformed): The interaction effect of serum uric acid and serum nickel on NAFLD when both UA and Ni are considered continuous variables (ln-transformed).

We performed a binary logistic regression analysis to investigate the association between SUA and serum Ni with NAFLD in Figure 2. The binary logistic regression models showed a significant association between SUA and serum Ni with the risk of NAFLD after the adjustment of confounding factors (*p* < 0.05). In Model 1, the risk of NAFLD significantly increased by 1.8 times (OR = 1.785, 95% CI: 1.314–2.428) with the high quartile when compared to the lowest quartile. In Model 2, the risk of NAFLD significantly increased by 1.6 times (OR = 1.579, 95% CI: 1.140–2.189) with the high quartile when compared to the lowest quartile. There was no statistical significance in the second and third quartiles of SUA (*p* > 0.05). The prevalence of NAFLD from the first quartile to the fourth was 35.70 (151/423), 44.60% (186/417), 43.29% (184/425), and 61.04% (271/444), respectively.

Similarly, in Model 1, the ORs (95% CI) for NAFLD from Ni_Q2 to Ni_Q4 were 1.340 (95% CI: 0.994–1.809), 1.406 (95% CI: 1.040–1.905), 2.137 (95% CI: 1.568–2.918), respectively. For Model 2, the ORs (95% CI) for NAFLD from Ni_Q2 to Ni_Q4 were 1.367 (95% CI: 1.003–1.865), 1.403 (95% CI: 1.024–1.923), 1.896 (95% CI: 1.372–2.652), respectively. When compared with the SUA model, the elevated concentrations of serum Ni had a higher risk of NAFLD than elevated concentrations of SUA in the serum Ni model. The prevalence of NAFLD from the first quartile to the fourth were 36.49 (154/422), 43.95% (189/430), 47.09% (202/429), and 57.94% (248/428), respectively. Surprisingly, when serum UA and serum Ni are considered as continuous variables (ln-transformed), after adjusting for all confounding factors, SUA and serum Ni had an interactive effect on NAFLD (OR = 2.327, 95% CI: 1.142–4.989, *p* = 0.024) based on a multiplicative model.

**Figure 3:**
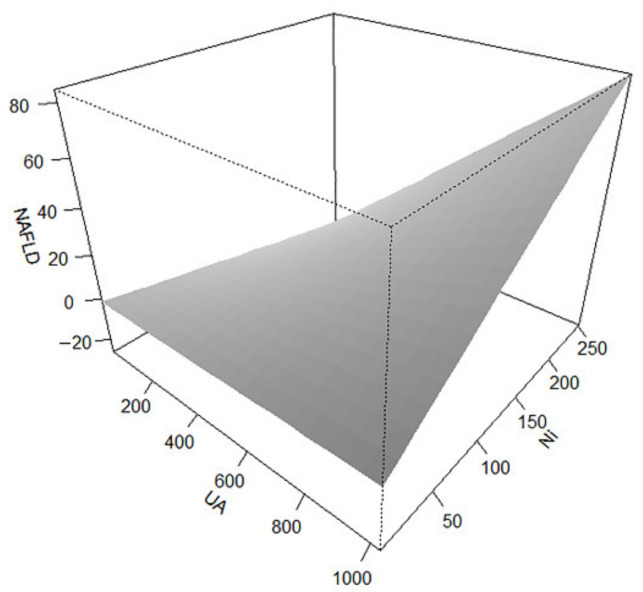
The interaction diagram shows SUA combining serum Ni with NAFLD. SUA and serum Ni are regarded as continuous variables.

Furthermore, in the interaction effect diagram, we can view that the SUA (continuous variables) combined with serum Ni (continuous variables) strengthen the risk of NAFLD (Figure 3).

**Figure 4:**
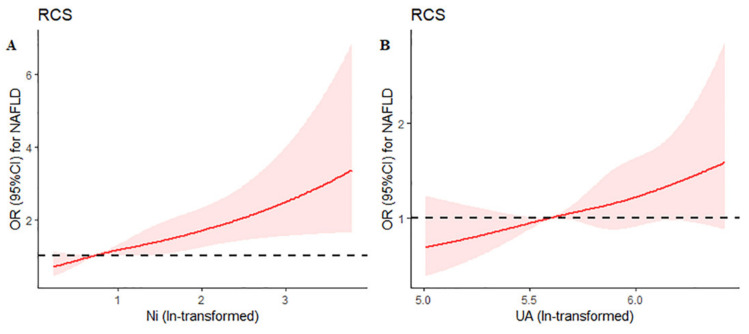
Restricted cubic spline analysis representing the associations between serum Ni (**A**) and UA (**B**) with NAFLD. Serum uric acid and nickel concentrations (ln-transformed, *X*-axis) and non-alcoholic fatty liver disease (*Y*-axis) were fitted by restricted cubic spline models with four knots at the 35th, 65th, and 95th percentiles of its distribution; the reference value (odds ratio = 1) was set at the 5th percentile. The red line represents the dose relationship between serum nickel (**A**) or serum uric acid (**B**) with the risk of NAFLD after logarithmic transformation. The black dotted line indicates odds ratio is 1, and the pink area indicates the confidence interval. Adjusted variables: age, sex, BMI SBP, DBP, AST, TBIL, ALP, TC, TG, LDL-C, HDL-C, fasting glucose, HbA_1C_.

Restricted cubic spline analysis indicated significant linear associations for serum Ni (ln-transformed) on NAFLD (*p* for overall < 0.001, *p* for non-linear = 0.808), while this similar relationship was not statistically significant for SUA (*p* for overall = 0.075, *p* for non-linear = 0.998) (Figure 4).

### 3.4. Comparison of SUA and Serum Ni in Lean NAFLD, Obese NAFLD, and Control Group

We further performed a subgroup analysis comparing NAFLD patients with different BMI to those without NAFLD in Figure S1. After adjusting for potential confounders, Ni increased the risk of lean NAFLD (OR = 1.019, 95% CI: 1.000–1.038, *p* = 0.045) and obese NAFLD (OR = 1.021, 95%CI: 1.003–1.040, *p* = 0.024) when compared with the control group; However, SUA (OR = 1.002, 95%CI: 1.000–1.003, *p* = 0.054) increased the risk of obese NAFLD at a statistically significant threshold, and was not associated with lean NAFLD. When compared with the lowest quartile, Ni increased the risk of lean NAFLD (OR = 2.646, 95%CI: 1.537–3.950, *p* < 0.001) and obesity NAFLD (OR = 1.975, 95% CI: 1.293–3.018, *p* = 0.002) in the highest quartile. However, when compared with the lowest quartile, SUA increased the risk of obesity NAFLD (OR = 1.823, 95% CI: 1.187–2.802, *p* = 0.006) in the highest quartile, null with lean NAFLD.

Restricted cubic spline analysis showed that serum Ni had a positive linear relationship with lean NAFLD (*p* for overall < 0.004, *p* for non-linear = 0.422) and obese NAFLD (*p* for overall = 0.003, *p* for non-linear = 0.926), and null SUA (*p* for overall > 0.05, *p* for non-linear > 0.05) in Figure S2.

### 3.5. Conjunctional Relationship of SUA and Serum Ni on NAFLD

**Figure 5:**
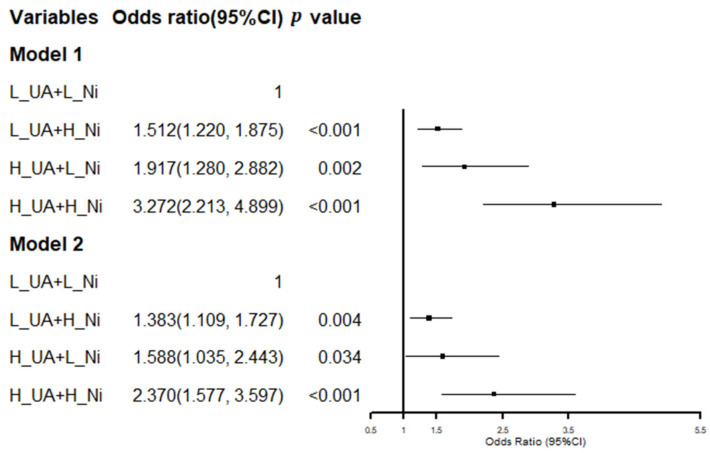
Conjunctional relationship of SUA and serum Ni on NAFLD. H_SUA ≥ 420 µmol/L; H_ Ni, high nickel, was defined as >2.53 µg/L; Model 1: adjusted for age and BMI; Model 2: further adjusted for SBP, DBP, ALT, AST, ALP, TBIL, TC, TG, LDL-C, HDL-C, fasting glucose, HbA_1C,_ meat consumption, and vegetable consumption.

Figure 5 displays a conjunctional relationship of SUA and serum Ni with NAFLD. When compared with those with L_SUA + L_Ni, individuals with L_UA + H_Ni (OR = 1.512, 95% CI: 1.220–1.875), H_UA + L_Ni (OR = 1.917, 95% CI: 1.280–2.882) and H_UA + H_Ni (OR = 3.272, 95% CI: 2.213–4.899) had an odds risk of NAFLD after adjustment for age and BMI (Model 1). After further adjustment for SBP, DBP, ALT, AST, ALP, TBIL, TC, TG, LDL-C, HDL-C, fasting glucose, HbA_1C_, meat consumption, and vegetable consumption, the ORs (95% CI) for NAFLD from L_UA + H_Ni to H_UA + H_Ni were 1.383 (95% CI: 1.109–1.727), 1.588 (95% CI: 1.035–2.443), and 2.370 (95% CI: 1.577–3.597), respectively, when compared to the L_SUA + L_Ni. In both models, SUA combined with Ni strengthens the risk of NAFLD more than SUA and Ni individually.

## 4. Discussion

The current study showed that the prevalence of NAFLD in males aged ≥18 years was 46.6%, with a mean serum nickel concentration of 2.5 µg/L. Our current study also showed a positive association between relatively high levels of SUA (in the fourth SUA quartile) and a positive linear association of serum Ni concentrations with NAFLD risk. Yet, a high serum Ni concentration was associated with a higher risk of NAFLD than high SUA concentration. More importantly, intensive combined effects of SUA and serum Ni with NAFLD risk were observed in our study. Further stratification of NAFLD subgroups, serum Ni was positively correlated with both lean and obese NAFLD, while SUA was only associated with obese NAFLD.

Our previous study reported that SUA level was independently and positively correlated with the prevalence of metabolic associated fatty liver disease, which is consistent with our current study [37]. Zheng X et al. [38] showed that SUA could be used as a marker to evaluate the severity of thin NAFLD. In addition, several studies have shown that increased SUA was closely associated with the risk of lean NAFLD [39,40], which is inconsistent with our study. This was attributed to the small number of lean people in our study, which needs to be verified by expanding the sample size and more prospective studies. An animal experiment further showed that acetate can abate liver lipids by inhibiting uric acid [41]. This implies that uric acid may have an important role in NAFLD by mediating lipid accumulation. Moreover, several population-based studies have indicated that SUA level was independently of NAFLD risk [42,43]. A prospective study also showed that SUA could be used to predict NAFLD [44]. Despite some studies reporting that elevated concentrations of SUA increased NAFLD risk in women higher than in men [45,46], the risk was still substantial in men. Yet, studies suggested that SUA increases NAFLD risk mainly in males [19,47]. All these above findings support that SUA is associated with NAFLD risk in men.

Previous studies revealed that Ni accumulation could damage the normal liver structure, and lead to hepatic fat accumulation, and hepatic steatosis [48,49]. Recent evidences suggests that high urine Ni (log per 1 µg/L; OR = 1.193 95% CI: 1.019–1.397) is associated with metabolic syndrome and lipid accumulation products [28]. A pilot cross-sectional analysis further showed a substantially higher prevalence of Ni allergy relevant to metabolic syndrome and fatty liver disease [50]. Besides, a case study from Chile revealed that low concentrations of urine Ni (median of 1.88 µg/L, with 25% of subjects’ urine concentrations above 2.55 µg/L) with hyperglycemia and elevated levels of the inflammatory mediator IL-6 were interrelated [51]. A study based on rice seeds demonstrated that high nickel levels could increase ROC production and lipid peroxidation through the oxidative stress response [52]. It is worth noting that insulin resistance induced lipid accumulation in hepatocytes and lipid-peroxidation-mediated inflammation may be the underlying pathological features of NAFLD [27]. Meanwhile, hyperuricemia is closely linked to insulin resistance and increased oxidative stress in the liver. It is also involved in the development and progression of NAFLD [15], and the toxic mechanism of Ni was also attributed to this [53] These findings are consistent with our current results, suggesting that serum Ni is a risk factor for NAFLD. In addition, animal studies suggested that Ni-activating lipid peroxidation and the overproduction of free radicals causes severe hepatotoxicity, renal toxicity, and cytotoxicity, which in turn leads to elevations of uric acid and elevations in the ASAT and ALAT activities in male rats [54,55,56,57]. Thus, the elevated concentrations of serum Ni would cause a higher risk of NAFLD than elevated concentrations of SUA.

To sum up, this study first reported the conjunctional relationship of SUA and serum Ni with NAFLD risk in men. Simultaneously, the combination of SUA and serum Ni reinforced the risk of NAFLD in men. Furthermore, this study suggested that lower serum Ni may be involved the pathogenesis of NAFLD. Finally, the physical examinations and laboratory measurements were standardized, and we implemented stricter criteria for inclusion. Yet, this study also has some limitations. To begin with, causality cannot be confirmed due to the current study being an observational study design. Then, results are not generalizable as the study was conducted in a single center, and included only one ethnic group and the male gender only. Furthermore, data regarding alcohol consumption were self-reported which may lead to bias. Finally, NAFLD was diagnosed using ultrasound methods instead of histological assessments.

## 5. Conclusions

Our study showed the existence of a positive association between SUA concentrations and serum Ni concentrations with the risk of NAFLD in men. Furthermore, the combined effect of SUA and Ni with NAFLD prevalence was reinforced in men.

## Figures and Tables

**Table 1:** The baseline characteristics.

Variables	Total (*n* = 1709)	NAFLD (*n* = 793)	Non-NAFLD (*n* = 916)	*p* ^3^
Prevalence (%)	46.4	100	0	
age (y) (median; IQR) ^2^	55 (45, 65)	53 (43, 64)	58.5 (47, 66)	<0.001
age (y) *n* (%) ^1^				<0.001
18 ≤ age ≤ 44	410 (24.0)	220 (53.7)	190 (46.3)	
45 ≤ age ≤ 59	573 (33.5)	284 (49.6)	289 (50.4)	
age ≥ 60	726 (42.5)	289 (39.8)	437 (60.2)	
BMI (kg/m^2^) *n* (%) ^1^				<0.001
BMI < 18.50	25 (1.6)	2 (8.0)	23 (92.0)	
18.50 ≤ BMI < 24	559 (32.6)	145 (25.9)	414 (74.1)	
BMI ≥ 24	1125 (65.8)	646 (57.4)	479 (42.6)	
SBP, mmHg (median; IQR) ^2^	129.7 (118.3, 143.0)	131.3 (119.7, 143.0)	128.0 (117.7, 142.7)	0.028
DBP, mmHg (median; IQR) ^2^	82.0 (74.7, 89.7)	82.7 (75.3, 91.0)	81.0 (73.9, 89.0)	0.001
ALT, U/L (median; IQR) ^2^	21.0 (15.5, 29.0)	23.4 (18.0, 33.0)	19.0 (14.0, 25.0)	<0.001
AST, U/L (median; IQR) ^2^	22.0 (19.0, 25.0)	22.0 (19.0, 26.0)	22.0 (19.0, 25.0)	0.450
ALP, U/L (median; IQR) ^2^	84.0 (71.0, 98.2)	82.0 (68.0, 96.0)	85.0 (72.0, 100.0)	0.001
TBIL, mmol/L (median; IQR) ^2^	16.8 (12.9, 22.2)	16.89 (13.0, 22.1)	16.7 (12.8, 22.2)	0.491
SUA, mmol/L (median; IQR) ^2^	323.0 (274.0, 380.6)	341.0 (289.0, 400.0)	310.0 (265.0, 360.0)	<0.001
Serum nickel, µg/L (median; IQR) ^2^	2.5 (2.0, 3.5)	2.8 (2.1, 4.0)	2.4 (1.9, 3.2)	<0.001
Total cholesterol, mg/dL (median; IQR) ^2^	5.0 (4.3, 5.6)	5.1 (4.4, 5.7)	4.9 (4.2, 5.5)	<0.001
Triglycerides, mg/dL (median; IQR) ^2^	1.4 (1.0, 2.0)	1.6 (1.1, 2.3)	1.2 (0.9, 1.7)	<0.001
LDL-C, mg/dL (median; IQR) ^2^	2.9 (2.4, 3.4)	3.0 (2.4, 3.6)	2.8 (2.3, 3.3)	<0.001
HDL-C, mg/dL (median; IQR) ^2^	1.2 (1.0, 1.3)	1.1 (1.0, 1.3)	1.2 (1.0, 1.4)	<0.001
Fasting glucose, mg/dL (median; IQR) ^2^	5.4 (5.1, 6.0)	5.6 (5.2, 6.2)	5.4 (5.0, 5.9)	<0.001
HbA_1C_, % (median; IQR) ^2^	5.6 (5.2, 5.9)	5.6 (5.2, 6.1)	5.5 (5.1, 5.9)	<0.001

Data are expressed as median (interquartile ranges) or numbers (proportions). BMI, Body mass index; SBP, Systolic blood pressure; DBP, Diastolic blood pressure; SUA, Serum uric acid; ALT, Alanine aminotransferase; ALP, Alkaline phosphatase; AST, Aspartate aminotransferase; TBIL, Total bilirubin; HbA_1C_, Glycosylated hemoglobin; HDL-C, High-density lipoprotein cholesterol; LDL-C, Low-density lipoprotein cholesterol; NAFLD, Non-alcoholic fatty liver disease. ^1^ Difference between NAFLD and non-NAFLD proportions was tested using the Chi-squared Test. ^2^ Difference of non-normally distributed data between NAFLD and non-NAFLD groups adopted the Mann–Whitney U test. ^3^ *p*-value refers to the comparison between NAFLD and non-NAFLD groups, *p*-value < 0.05 indicates statistical significance.

## Data Availability

The datasets used for the current study are available from the corresponding author on reasonable request.

## Supplementary Materials

The following supporting information can be downloaded at: https://www.mdpi.com/article/10.3390/ijerph19116424/s1. Supplementary Tables. Table S1. Basic characteristics according to the serum uric acid quartile (median; IQR). Table S2. Basic characteristics according to the nickel quartile (median; IQR). Supplementary Figures. Figure S1. Comparison of SUA and serum Ni in lean NAFLD, obese NAFLD, and control group. Figure S2. Restricted cubic spline analysis the associations between serum Ni (A) and serum UA (B) with lean NAFLD, and the associations between serum Ni (C) and serum UA (D) with obese NAFLD.
