# Supplementary material for: Conjunctional Relationship between Serum Uric Acid and Serum Nickel with Non-Alcoholic Fatty Liver Disease in Men: A Cross-Sectional Study
Source: Int J Environ Res Public Health. 2022 May 25;19(11):6424. doi: 10.3390/ijerph19116424 (PMC9180290; doi:10.3390/ijerph19116424)
Supplement: Supplementary file 1 [file ijerph-19-06424-s001.zip › ijerph-1711639-supplementary.pdf]

Supplementary Tables

Table S1. *Basic characteristics according to the serum uric acid quartile (median; IQR)*

**Variables**	**SUA_Q1 < 275μmol/L (n = 423)**	**SUA_Q2 275-322μmol/L (n = 417)**	**SUA_Q3 322-379μmol/L (n = 425)**	**SUA_Q4 ≥ 379μmol/L (n = 444)**	***P***
Age (median; IQR)	61.0(50.0, 67.0)	59.0(49.0, 66.0)	53.0(43.0, 64.0)	48.0(36.0, 62.3)	<0.001
BMI (median; IQR)	24.2(22.4, 26.5)	25.3(23.1, 27.1)	25.6(23.4, 27.6)	26.8(24.2, 29.1)	<0.001
SBP, mmHg (median; IQR)	129.7(118.8, 142.8)	130.7(118.7, 143.0)	129.7(117.3, 143.7)	128.7(119.0, 141.7)	0.899
DBP, mmHg(median; IQR)^2	80.7(73.7, 88.3)	81.3(75.0, 88.3)	82.7(74.7, 90.3)	83.0(75.3, 91.0)	0.059
Total cholesterol, mg/dl (median; IQR)	4.9(4.4, 5.5)	5.0(4.3, 5.5)	4.9(4.3, 5.6)	5.2(4.4, 5.9)	0.011
Triglycerides, mg/dl (median; IQR)	1.2(0.8, 1.7)	1.3(1.0, 1.7)	1.4(1.0, 2.0)	1.7(1.2, 2.5)	<0.001
LDL-C, mg/dl (median; IQR)	2.8(2.3, 3.3)	2.9(2.4, 3.4)	2.9(2.4, 3.5)	3.0(2.4, 3.6)	0.101
HDL-C, mg/dl (median; IQR)	1.2(1.0, 1.4)	1.2(1.0, 1.4)	1.1(1.0, 1.3)	1.1(0.9, 1.3)	<0.001
ALT, U/L(median; IQR)	19.0(14.0, 27.0)	20.0(15.0, 27.0)	21.0(16.0, 29.0)	23.5(17.0, 34.0)	<0.001
AST, U/L(median; IQR)	22.0(19.0, 25.0)	22.0(19.0, 26.0)	21.0(19.0, 24.0)	22.8(20.0, 26.0)	0.005
ALP, U/L (median; IQR)	85.0(73.0, 103.0)	84.7(71.0, 97.0)	84.0(70.0, 97.0)	81.0(70.0, 96.0)	0.038
TBIL, mmol /L (median; IQR)	16.4(13.0, 22.0)	16.7(12.9, 21.7)	16.9(13.0, 22.8)	17.0(12.8, 22.4)	0.738
SUA (mmol/L)	243.0(217.3, 261.5)	299.4(287.0, 310.0)	346.0(334.0, 361.0)	430.0(398.0, 467.3)	<0.001
Serum nickel, μg/L	2.5(2.0, 3.4)	2.5(2.0, 3.5)	2.5(2.1, 3.3)	2.8(2.1, 3.9)	<0.001
Fasting glucose, mg/dl(median; IQR)	5.5(5.1, 6.4)	5.4(5.1, 6.1)	5.4(5.0, 5.9)	5.5(5.1, 5.9)	0.054
HbA_{1c}, % (median; IQR)	5.6(5.2, 6.1)	5.6(5.2, 6.0)	5.5(5.1, 5.9)	5.5(5.1, 5.8)	<0.001

Data are expressed as median (interquartile ranges) or numbers (proportions); BMI, Body mass index; SBP, Systolic blood pressure; DBP, Diastolic blood pressure; SUA, Serum uric acid; ALT, Alanine aminotransferase; ALP, Alkaline phosphatase; AST, Aspartate aminotransferase; TBIL, Total bilirubin; HbA_{1c}, Glycosylated hemoglobin; HDL-C, High-density lipoprotein cholesterol; LDL-C, Low-density lipoprotein cholesterol. The difference in non-normally distributed data were analyzed by the Mann-Whitney U test; *P*-value < 0.05 indicates statistical significance.

Table S2. *Basic characteristics according to the nickel quartile (median; IQR)*

Variables	Ni_Q1<br>< 2.03μg/L<br>(n = 422)	Ni_Q2<br>2.03-2.53μg/L<br>(n = 430)	Ni_Q1<br>2.53-3.53μg/L<br>(n = 429)	Ni_Q4<br>≥ 3.53 μg/L<br>(n = 428)	*P*
Age (median; IQR)	63.0(53.3, 68.0)	60.0(46.0, 67.0)	54.0(43.0, 64.0)	48.0(41.0, 56.3)	<0.001
BMI (median; IQR)	25.2(22.7, 27.4)	25.1(23.0, 27.7)	25.6(23.2, 27.8)	25.9(23.5, 28.0)	0.026
SBP, mmHg (median; IQR)	131.3(120.0, 147.0)	130.8(119.0, 143.0)	131.0(119.7, 145.3)	126.0(116.7, 137.4)	<0.001
DBP, mmHg (median; IQR)	82.3(75.0, 90.9)	81.7(74.4, 89.3)	82.0(75.0, 90.7)	81.3(74.7, 88.3)	0.37
Total cholesterol, mg/dl (median; IQR)	5.0(4.4, 5.6)	4.9(4.3, 5.5)	5.0(4.2, 5.7)	5.0(4.4, 5.6)	0.127
Triglycerides, mg/dl (median; IQR)	1.3(0.9, 1.8)	1.3(1.0, 1.9)	1.4(1.0, 1.9)	1.5(1.1, 2.2)	<0.001
LDL-C, mg/dl (median; IQR)	2.9(2.4, 3.3)	2.9(2.4, 3.5)	2.9(2.3, 3.5)	2.9(2.4, 3.4)	0.926
HDL-C, mg/dl (median; IQR)	1.2(1.0, 1.4)	1.2(1.0, 1.4)	1.1(1.0, 1.3)	1.1(0.9, 1.3)	<0.001
ALT,U/L(median; IQR)	19.0(14.8, 26.0)	20.7(16.0, 28.0)	20.0(15.0, 30.0)	22.0(16.6, 32.0)	<0.001
AST,U/L(median; IQR)	22.0(19.0, 26.0)	22.0(19.0, 25.0)	22.0(19.0, 25.0)	22.0(19.0, 25.1)	0.795
ALP, U/L(median; IQR)	85.0(70.9, 101.0)	84.0(72.0, 97.0)	85.0(70.0, 100.0)	81.0(70.0, 95.0)	0.186
TBIL, mmol /L (median; IQR)	16.3(13.0, 22.3)	16.1(12.3, 21.3)	17.8(13.7, 22.7)	17.1(12.9, 22.1)	0.061
SUA (mmol/L)	310.7(266.0, 366.5)	320.0(273.0, 376.5)	328.0(275.0, 382.0)	335.0(278.8, 399.2)	<0.001
Serum nickel, μg/L	1.7(1.6, 1.9)	2.3(2.2, 2.4)	2.9(2.7, 3.2)	5.3(4.2, 7.6)	<0.001
Fasting glucose, mg/dl(median; IQR)	5.4(5.1, 6.2)	5.5(5.1, 6.0)	5.5(5.1, 6.0)	5.4(5.0, 5.9)	0.542
HbA_{1c}, % (median; IQR)	5.7(5.2, 6.1)	5.6(5.2, 5.9)	5.5(5.1, 5.9)	5.4(5.1, 5.8)	<0.001

Data are expressed as median (interquartile ranges) or numbers (proportions); BMI, Body mass index; SBP, Systolic blood pressure; DBP, Diastolic blood pressure; SUA, Serum uric acid; Ni, nickle; ALT, Alanine aminotransferase; ALP, Alkaline phosphatase; AST, Aspartate aminotransferase;TBIL, Total bilirubin; HbA_{1c}, Glycosylated hemoglobin; HDL-C, High-density lipoprotein cholesterol; LDL-C, Low-density lipoprotein cholesterol. The difference in non-normally distributed data were analyzed by the Mann-Whitney U test; *P*-value < 0.05 indicates statistical significance

Supplementary Figures

Group	Number	Odds ratio(95%CI)	P value
Control	n=916		
lean-NAFLD	n=224		
SUA_Q1		1	
SUA_Q2		0.999(0.637, 1.565)	0.996
SUA_Q3		1.082(0.706, 1.660)	0.717
SUA_Q4		1.319(0.828, 2.101)	0.243
UA		1.001(0.999, 1.003)	0.229
Ni_Q1		1	
Ni_Q2		1.510(0.958, 2.381)	0.076
Ni_Q3		1.510(0.937, 2.432)	0.091
Ni_Q4		2.464(1.537, 3.950)	<0.001
Ni		1.019(1.000, 1.038)	0.045
obese-NAFLD	n=569		
SUA_Q1		1	
SUA_Q2		1.247(0.816, 1.904)	0.307
SUA_Q3		1.732(1.142, 2.626)	0.010
SUA_Q4		1.823(1.187, 2.802)	0.006
UA		1.002(1.000, 1.003)	0.054
Ni_Q1		1	
Ni_Q2		1.372(0.909, 2.072)	0.132
Ni_Q3		1.544(1.025, 2.328)	0.038
Ni_Q4		1.975(1.293, 3.018)	0.002
Ni		1.021(1.003, 1.040)	0.024

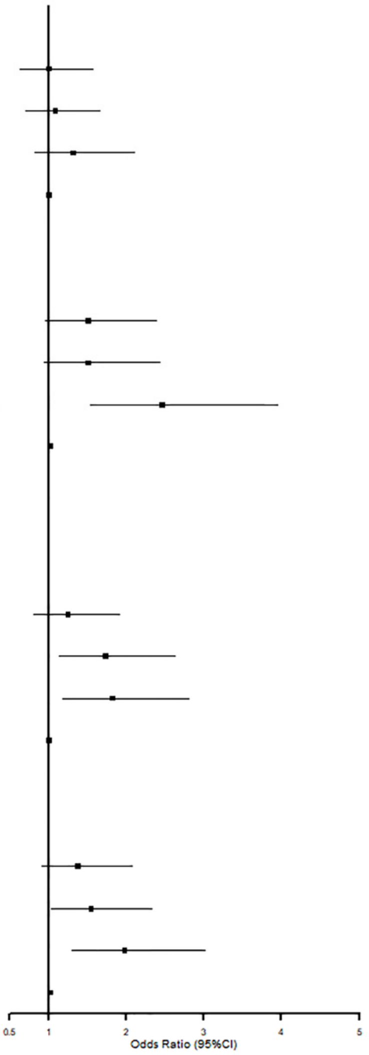

Figure S1 Comparison of SUA and serum Ni in lean NAFLD, obese NAFLD and control group. The SUA quartiles were as follows: < 275, 275-322, 322-379 and ⩾ 379 μmol/L. The serum Ni quartiles were as follows: < 2.03, 2.03-2.53, 2.53-3.53 and ⩾ 3.53 μg/L. Model 1: adjusted for age and BMI; Model 2: further adjusted for SBP, DBP, ALT, AST, ALP, TBIL, TG, LDL-C, HDL-C, fasting glucose, HbA_{1C}, meat consumption and vegetables consumption.

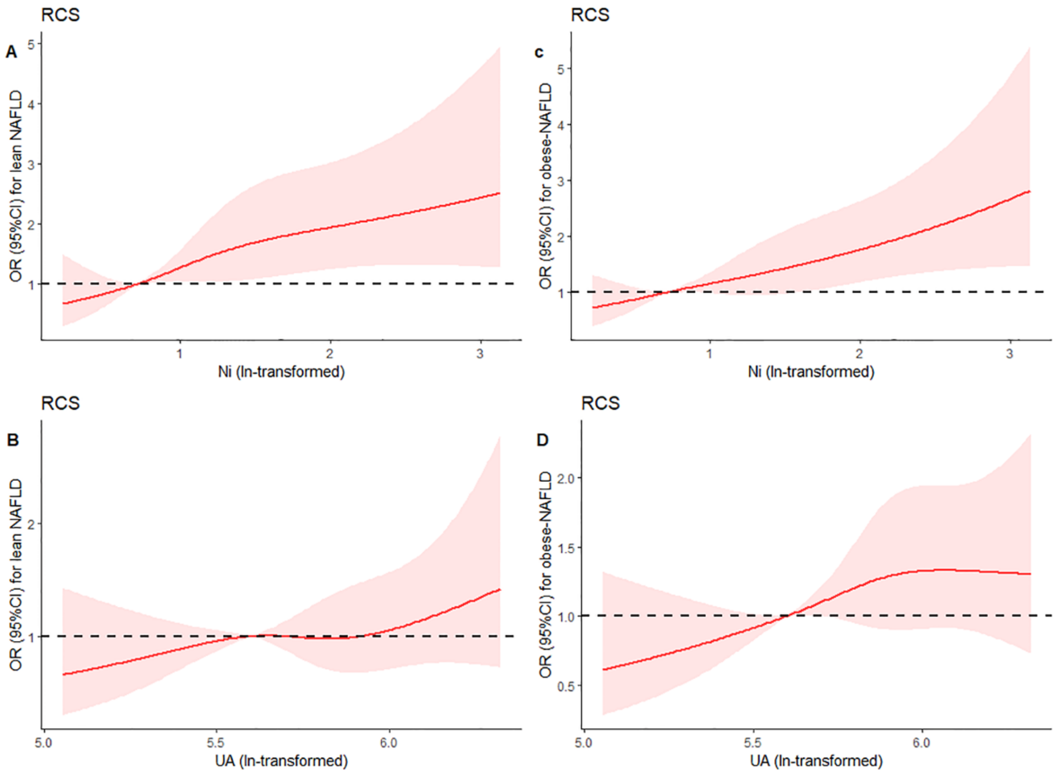

Figure S2. Restricted cubic spline analysis representing the associations between serum Ni (A) and UA (B) with lean NAFLD, and between serum Ni (C) and UA (D) with obese NAFLD. Serum uric acid and nickel concentrations (ln-transformed, x-axis) and lean NAFLD or obese NAFLD (y-axis) were fitted by restricted cubic spline models with four knots at the 35th, 65th and 95th percentiles of its distribution; the reference value (odds ratio =1) was set at the 5th percentile. The red line represents the dose relationship between serum nickel or serum uric acid with the risk of lean NAFLD or obese NAFLD after logarithmic transformation. The black dotted line indicates odds ratio is 1, and the pink area indicates the confidence interval. Adjusted variables: age, sex, BMI SBP, DBP, AST, TBIL, ALP, TC, TG, LDL-C, HDL-C, fasting glucose, HbA_{1C}.
